# The Wound Healing: A Mystery Still to Be Solved—What Is the Future?

**DOI:** 10.3390/biomedicines14040926

**Published:** 2026-04-18

**Authors:** Montserrat Fernández-Guarino, María Luisa Hernández Bule, Stefano Bacci

**Affiliations:** 1 Dermatology Service, Hospital Ramón y Cajal, Instituto Ramón y Cajal de Investigación Sanitaria (Irycis), 28034 Madrid, Spain; drafernandezguarino@gmail.com; 2 Department of Preclinical Dentistry I, Faculty of Biomedical and Health Sciences, European University of Madrid, 28670 Madrid, Spain; marialuisa.hernandez@universidadeuropea.es; 3 Research Unit of Histology and Embriology, Department of Biology, University of Florence, Viale Pieraccini 6, 50134 Firenze, Italy

**Keywords:** acute wounds, cellular infiltrate, chronic wounds, keloids, scars

## Abstract

This perspective contains the current understanding of the cellular and molecular mechanisms involved in wound healing (the articles taken into consideration relate to the three-year period 2023–2025). Nevertheless, these biological pathways remain inadequately characterized; this is seen by the modifications leading to pathological conditions, such as keloids, chronic wounds, or hypertrophic scars and diabetic wounds. Focus is also directed to novel therapy suggested for these types of conditions. Understanding these scientific issues is crucial for professionals across many fields who see such presentations often.

## 1. Introduction

Wound healing (WH) is a significant public health issue. In the United States, millions of people are affected by chronic wounds (CWs) [1], and costs are expected to increase due to variables such as an aging population and rising rates of diabetes. Furthermore, estimates suggest that over 1.5 million people in Europe suffer from CWs. This situation has led to an increase in specialty programs for the training of medical professionals such as primary care physicians, vascular surgeons, nurses, and dermatologists. A complete understanding of the biological principles underlying wound healing, however, is still lacking, despite the clear need for innovation in wound healing technologies, which are critical for improving patient outcomes and reducing the burden of conditions like CWs; this gap in knowledge contributes to the ongoing challenges faced by medical professionals in effectively treating patients with chronic wounds. Current debates often focus on specialized topics without sufficient interdisciplinary cooperation, which hinders progress in understanding wound healing as a complex process that requires collaboration across various medical fields, such as integrating insights from immunology, genetics, and surgical techniques. Based on the above, wound healing still remains a mystery to be solved. Attempts in this direction, such as neuro-immune integration, regeneration versus scarring, or genetic variability, have been made, however, as discussed in the following paragraphs [1,2,3].

## 2. Significant Occurrences in WH (In Brief)

**Figure 1:**
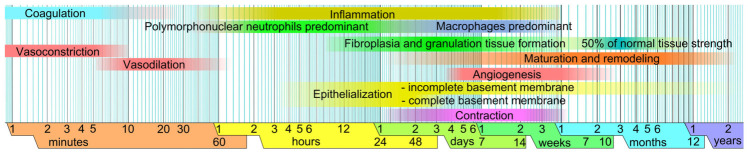
Different phases of wound healing. By Mikael Häggström. When using this image in external works, it may be cited as: Häggström, Mikael (2014). “Medical gallery of Mikael Häggström 2014”. WikiJournal of Medicine 1 (2). DOI:10.15347/wjm/2014.008. ISSN 2002-4436. Public Domain or By Mikael Häggström, used with permission. Own work (from the template Logarithmic time scale—milliseconds to years.svg), Public Domain, https://commons.wikimedia.org/wiki/File:Wound_healing_phases.svg (accessed on 27 February 2026).

WH occurs through four overlapping phases: hemostasis, inflammation, proliferation, and remodeling/maturation [1,2,3] [Figure 1].

During the hemostasis phase, endothelial cells release the von Willebrand factor (VWF), enhancing platelet adhesion, leading to clot formation and the cessation of bleeding. This process is facilitated by smooth muscle contraction due to increased calcium ion levels, resulting in vessel narrowing and reduced blood flow. This phase typically lasts a few minutes [1,2,3].

The inflammatory phase begins next, with mast cells (MCs) releasing histamine or serotonin to cause vasodilation. Neutrophils and monocytes migrate to the wound site, with vimentin playing a crucial role in mediating their attachment and movement. This phase, lasting 0–3 days, is characterized by phagocytosis of pathogens and damaged cells, fueled by cytokines and growth factors secreted by leukocytes, as well as inflammatory cytokines from keratinocytes [1,4]. Studies indicate that inflammatory cells begin undergoing apoptosis within 12 h following injury, a critical event in the inflammatory phase of skin wound healing. Neutrophils are among the first responders, migrating to the injury site to phagocytose pathogens and produce neutrophil extracellular traps (NET). Following their active role, neutrophils undergo apoptosis and are predominantly efferocytosed by macrophages [5,6]. Efferocytosis is a highly coordinated, multi-stage process designed to remove dead cells silently, maintaining tissue homeostasis. When this tightly regulated machinery fails—often due to dysregulation of key molecular switches like Vav3, MerTK, or LRP1—the resulting accumulation of secondary necrotic cells leads to chronic inflammation and, of course, problems during wound healing [7]. The rapid removal of apoptotic neutrophils is crucial for resolving inflammation, as it inhibits the synthesis of pro-inflammatory cytokines and facilitates the release of beneficial factors such as transforming growth factor (TGF) β1 and prostaglandin E2 (PGE2), both of which are essential for effective wound closure [5,6]. However, in instances where macrophages lack the guanine-nucleotide exchange factor Vav3, the phagocytosis of apoptotic neutrophils is hindered. This impairment leads to a decreased release of TGFβ1, resulting in insufficient myofibroblast differentiation and ultimately delaying the processes of wound contraction and closure and tissue remodeling [7].

In the proliferative phase, which lasts 3–12 days, fibroblasts contribute to granulation tissue formation while regulating keratinocyte dynamics and angiogenesis [1,2,3,4]. Macrophages and MCs also secrete growth factors, but vimentin deficiency at this stage can hinder fibroblast proliferation, TGFβ production, collagen synthesis, and epithelial re-epithelialization [8]. Apoptosis of immune cells and their subsequent efferocytosis sets off the proliferative and migratory phase of keratinocytes, eliciting re-epithelialization in the healing wound edge to restore the epidermal barrier [5,6].

The remodeling/maturation phase involves collagen restoration and wound contraction, primarily driven by myofibroblasts. Growth factors regulate mesenchymal transformations through TGF-β and Notch signaling pathways, which impact endothelial cell behavior. This phase can extend from 3 days to 6 months, culminating in scar formation through the remodeling of granulation tissue, with matrix metalloproteinases (MMPs) and their inhibitors (TIMPs) playing integral roles [1,4]. The extracellular matrix changes, with type III collagen taking the place of type I collagen and elastin coming back. Some cells in the granulation tissue die as the wound heals, and there is still debate about where the fibrocytes in the dermis come from during these processes. The process of apoptosis of myofibroblasts, which occurs during the remodeling phase, is responsible for promoting wound closure by initiating the transformation of granulation tissue into scar tissue. On the other hand, significant inhibition of myofibroblast efferocytosis can also lead to hypertrophic scar formation and fibrosis [5,6].

## 3. Current Concepts in Understanding the Key Cells Involved in Wound Healing

### 3.1. Phase: Hemostasis

#### Platelets

Platelets, non-nucleated fragments originating from bone marrow megakaryocytes, have crucial roles in physiological processes upon activation. During homeostasis, their activation results in the synthesis and release of several cytokines and growth factors, such as Platelet-Derived Growth Factor (PDGF), TGF-beta, and Vascular Endothelial Growth Factor (VEGF). These factors are essential to clot formation and functionality via many pathways: endocrine, paracrine, autocrine, and intracrine. In addition to hemostasis, platelet activity is essential for wound healing, promoting the migration and proliferation of fibroblasts, the differentiation of endothelial cells into new blood vessels, and the transformation of MSCs into specialized cell types. PDGF stimulates neutrophils and macrophages while also acting as a mitogenic and chemoattractant factor for fibroblasts and smooth muscle cells. In addition, in reaction to tissue injury, platelets initiate the inflammatory response by releasing prostaglandins, leukotrienes, and thromboxanes, underscoring their multifaceted involvement in vascular biology and tissue healing processes [1,3].

### 3.2. Phase: Inflammatory

#### 3.2.1. Mast Cells

The examination of MCs near the periphery of a skin lesion indicates substantial changes in their quantity and functionality over time. Within three hours after the injury, both the quantity of mast cells and their degranulation index exhibit an increase. Nonetheless, these values diminish below baseline levels after 6 h and then recuperate over the ensuing 10 days, returning to control values by 21 days. The first rise in MCs is probably linked to the elevated production of Monocyte Chemoattractant Protein (MCP) 1, which promotes the release of TGFbeta, a crucial chemoattractant for MCs [9,10].

Mast cells also facilitate hemostasis by secreting TNF alpha, which increases the production of Factor XIIIa in dermal dendrocytes, therefore stabilizing blood clots. They also secrete several inflammatory mediators, including histamine, VEGF, interleukins (ILs) such as IL6 and IL8, and TNFalpha, which enhance endothelial permeability and promote the migration of monocytes and neutrophils toward the wound. The secretion of leukotrienes, proteases, and cytokines by MCs subsequently generates chemotactic signals for granulocytes, whereas tryptase and cathepsin G are essential for endothelial-leukocyte interactions [11].

Regarding angiogenesis, TGF-beta promotes vascular development, but the compounds generated by MCs, such as VEGF, chymase, tryptase, and heparin, may paradoxically impede angiogenesis via interactions with anti-angiogenic proteins, which can lead to impaired healing and tissue regeneration in certain contexts.

Furthermore, throughout the maturation and remodeling stages of healing, MCs stimulate fibroblasts to augment collagen production, a process largely mediated by tryptase. MCs also produce growth factors and cytokines that affect the development of fibroblasts into myofibroblasts and release MMPs that facilitate the destruction of the extracellular matrix, aiding in tissue remodeling [9].

Alongside the conventional activation of resident receptors by the release of local mediators, emerging evidence supports the role of exosomes in intercellular communication, facilitating the transfer of particular cargo to influence recipient cell activity. MC exosomes have been identified as an alternative source of pro-fibrotic agents and represent a distinct mechanism for the production of surplus collagen produced by fibroblasts [12]. In addition, a large body of research has looked at how different components of exosomes released by mesenchymal stem cells (MSCs) affect MC. Evidence suggests that MSC exosomes may suppress cutaneous MC growth in Toll-Like Receptor (TLR)7 agonist-treated mice. Furthermore, it has been shown that TNFalpha-stimulated MSC medium reduces symptoms of experimental allergic conjunctivitis by inhibiting MC activation and histamine release via a COX2-dependent mechanism [13].

#### 3.2.2. Neutrophils

Neutrophils swiftly reach wound sites during the first hour after damage and gradually permeate the region due to diverse signaling molecules. Prominent among them are PDGF and Connective Tissue Chemokine-Activating Peptide (CTAPIII), which are secreted by platelets stimulated by Neutrophil-Activating Peptide 2 (NAP2) and CXCL7 in neutrophils. Notably, decreased levels of NAP2 facilitate neutrophil chemoattraction via the CXCR2 receptor; moreover, Growth-Related Oncogene (GRO) alpha produced by endothelial cells and pericytes amplifies this migratory response. Neutrophil recruitment is enhanced by ENA78 (CXCL5) and is linked to the activity of several signaling and chemotactic agents, including damage-associated molecular patterns (DAMPs) from necrotic cells, as well as molecules such as TGFbeta, C3a, C5a, and hydrogen peroxide generated by platelets. DAMPs interact with neutrophils by attaching to certain Pattern Recognition Receptors (PRRs), such as TLRs, and by promoting the secretion of supplementary chemoattractants [1,2,3].

In this situation, CXCL1, CXCL2, and CXCL8 are essential for the recruitment of inflammatory cells. Upon accumulation at the injury site, neutrophils release pro-inflammatory mediators including TNFalpha, IL1beta, IL6, and CXCL8, hence amplifying the inflammatory response. Neutrophils are essential in avoiding wound infections by eradicating bacteria and generating an oxidized environment via the formation of reactive oxygen species (ROS). They also secrete antimicrobial proteins and use chromatin traps and proteases to eliminate surrounding bacteria [1,2,3].

Neutrophil heterogeneity has recently been revealed to be present in the presence of diverse subtypes that display different functional states. These subtypes contribute in their own unique way to the various stages of innate immunity and wound healing. The local microenvironment, the presence of certain cytokines, and the kind of damage are all factors that might contribute to the development of this heterogeneity over time leading to variations in neutrophil responses that are crucial for effective innate immunity and wound healing The many functional states of neutrophils make it possible for them to respond to stress and damage in a manner that is precisely calibrated, which is required for the process of healing to be successful [14].

Recent research underscores the intricate interactions of neutrophil extracellular traps (NETs) with critical skin cell types such as macrophages, fibroblasts, keratinocytes, and endothelial cells, which are essential for regulating the acute inflammatory response, maintaining skin barrier integrity, and modulating angiogenesis [15].

Neutrophils facilitate angiogenesis and the proliferation of fibroblasts and keratinocytes by stimulating the production of cytokines (e.g., IL1beta and IL8), growth factors (e.g., VEGF), and chemokines (e.g., MCP1). The presence of neutrophils near the lesion diminishes after 24 to 36 h due to apoptosis, coinciding with the inflow of circulating monocytes attracted by IL8 from the neutrophils, which subsequently develop into macrophages, therefore advancing the healing process [1,2,3].

#### 3.2.3. Macrophages

Macrophages are essential in wound healing, especially during the inflammatory and proliferative stages, since they are attracted to injury sites within 48 to 72 h by chemical signals from diverse cell types. Their granules include significant growth factors, including TGFbeta and Epidermal Growth Factor (EGF), which play roles in inflammation, angiogenesis, and granulation tissue development. Macrophages undergo differentiation into two kinds throughout this process: M1 and M2. The M1 subtype, stimulated by TLR4 and Interferon gamma, operates in a pro-inflammatory manner, while the M2 subtype arises under the influence of IL4 and/or IL13, resulting in the secretion of supplementary growth factors, such as PDGF and Fibroblast Growth Factor (FGF). FGFbeta functions as a chemotactic and mitogenic agent, facilitating the proliferation of many cell types associated with angiogenesis [1,2,3].

Research indicates that the particular macrophage subtypes engaged in healing are dictated by alterations in macrophage morphologies, which are defined by unique surface markers and the secretion of cytokines, growth factors, and chemokines. Exosomes have garnered interest for their function in intercellular communication, since they are recognized for modulating macrophage polarization and aiding the shift from M1 to M2 phenotypes. This transition is essential for mitigating inflammatory responses and promoting tissue regeneration; however, the mechanisms by which exosomes affect macrophage alterations in diabetic wound contexts are mostly unexamined [16].

In the proliferative phase of healing, macrophages are strategically located near emerging blood vessels, promoting their differentiation by secreting IL-1, which encourages endothelial cell proliferation and angiogenesis. Alongside FGFbeta, other growth factors and cytokines, including TNF, IL6, and IL1, play a role in collagen synthesis, wound contraction, and epithelialization. Macrophages, in conjunction with keratinocytes and endothelial cells, synthesize MCP1, which recruits monocytes, MC, and lymphocytes during WH and facilitates endothelial cell migration during angiogenesis. During the remodeling phase, macrophages secrete matrix MMPs to dismantle the provisional extracellular matrix and eventually undergo apoptosis, signifying the conclusion of their essential function in the healing process [1,2,3].

***Involvement of other cells.***

#### 3.2.4. Dendritic Cells

Intercellular contacts between dendritic cells (DCs) and MCs are pivotal in the release of soluble factors, including TGFbeta and TNFalpha, which are crucial for the differentiation of precursor cells into DCs. Observations reveal that DCs are seldom seen in wounds less than one day old; however, their increase is observed in wounds aged between three and fourteen days. A unique subset of DCs in the human epidermis expresses CD1a and CD207 (langerin), referred to as Langerhans cells. Studies have shown that the quantity of these cells climbs significantly during the first hour post-injury. Besides utilizing a mouse model, it has been demonstrated that the absence of DCs impedes complete wound healing, thereby highlighting their critical function in the early phases of wound repair through the secretion of factors that stimulate the proliferation of healing-associated cells [1].

#### 3.2.5. Plasmacytoid Dendritic Cells

Plasmacytoid dendritic cells (PDCs) are essential to the immune response, since they generate type I interferon (IFN) and express TLR7 and TLR9. PDCs are not present in healthy skin but are rapidly recruited to damaged areas. Type I interferon is released as a consequence of their recruitment via interactions with regulatory T cells (Tregs). This IFN production is crucial since it facilitates wound healing, underscoring the significance of PDCs in tissue repair processes post-injury [1].

#### 3.2.6. Lymphocytes

Lymphocytes reach the lesion site 72 h after damage, drawn by Interleukin 1 (IL-1), released by macrophages, which is essential for extracellular matrix remodeling. Together with the involvement of antigen-presenting cells, factors like IFNgamma and TNFalpha inside the skin’s milieu initiate the lymphocyte response. Upon encountering the lesion, lymphocytes initiate the secretion of chemokines, commencing with Monocyte Chemoattractant Protein 1 (MCP-1), followed by Interferon gamma-Inducible Protein 10 (IP-10, also referred to as CXCL10), Monokine induced by gamma interferon (MIG; CXCL9), and Macrophage-Derived Chemokine (MDC; CCL22) after a period of four days. Lymphocytes also release growth factors, including EGF and FGF-beta. Recent findings underscore the significance of Tregs in cutaneous wound healing via the enhancement of EGF receptor expression. The lack of EGF in Tregs impedes their activation and migration, causing an accumulation of pro-inflammatory macrophages and protracted wound healing [1,2,3]. Tregs are essential in wound healing because they suppress pro-inflammatory cells and lessen tissue damage via the secretion of anti-inflammatory cytokines (IL10, TGFβ). Important for debris clearance and repair initiation, they mediate the change in macrophage phenotypes from inflammatory (M1) to pro-healing (M2). Tregs also promote angiogenesis and stem cell proliferation via the secretion of growth factors such as amphiregulin (AREG), IL22, and Insulin-Like Growth Factor 1 (IGF1). Additionally, they facilitate interactions unique to different tissues, which activate stem cells in the skin and help with hair follicle regeneration. In order to facilitate efficient wound healing, Tregs aid in immunological homeostasis by directing the progression from inflammation to proliferation and remodeling [17,18].

#### 3.2.7. Mesenchymal Stem/Stromal Cells (MSCs)

Mesenchymal stem/stromal cells (MSCs) are multipotent cells that may differentiate into specific cell types, including osteocytes, chondrocytes, and adipocytes. These cells are found in several adult and neonatal organs, including bone marrow, adipose tissue, and umbilical cords. MSCs possess essential traits such as self-renewal capability and considerable differentiation potential. Furthermore, they have significant immunomodulatory capabilities, enabling them to affect and diminish immunological responses. MSCs facilitate tissue repair and regeneration via many ways; they enhance angiogenesis, the development of new blood vessels, and release an array of advantageous chemicals that support healing processes [19,20].

Exosomes derived from mesenchymal stem cells (MSCs) have shown efficacy in facilitating wound healing via the enhancement of epithelialization, collagen maturation, and scar reduction. Human adipose-derived stem cells (hASCs) and their exosome-based derivatives have attracted interest for their capacity to expedite wound healing via many mechanisms, including the control of inflammation and the enhancement of cell proliferation and migration [21,22]. A critical analysis underscores the need for further investigation into the specific mechanisms of action of extracellular vesicles (EVs), their cargo composition, and their selective cellular targets in the wound microenvironment. Although EVs are known to expedite wound healing by modulating inflammatory, proliferative, and remodeling phases, the underlying signaling pathways and their regulation during different phases are not fully delineated. Understanding receptor–ligand dynamics is crucial as it remains unclear how EVs selectively target cells, leading to questions about true biological targeting versus random accumulation in damaged tissues. The “therapeutic cargo” of EVs, including proteins, lipids, and nucleic acids, is complex and varies based on the source and culture conditions. Standardized methods for quantifying active cargo are lacking, complicating dosage determinations. The dynamic wound microenvironment, populated by inflammatory cells, fibroblasts, and keratinocytes, necessitates a clear understanding of EV targets. EVs are known to mediate macrophage M1-to-M2 polarization, but the specifics of cargo-induced transitions in chronic inflammation are not well characterized. Furthermore, the assumption that systemically administered EVs will reach their targets is challenged by the inflammatory and hypoxic environments of chronic wounds. The involvement of EVs in nerve regeneration and their interaction with Schwann cells is an emerging area, yet it is under-researched concerning cutaneous nerve repair. A shift from simply stating therapeutic potential to a mechanism-focused approach is warranted, addressing challenges such as short half-lives and low concentrations of active EVs, standardizing isolation techniques, and validating mechanisms in more complex models before clinical implementation [23].

### 3.3. Phase: Proliferation

#### 3.3.1. Keratinocytes

**Figure 2:**
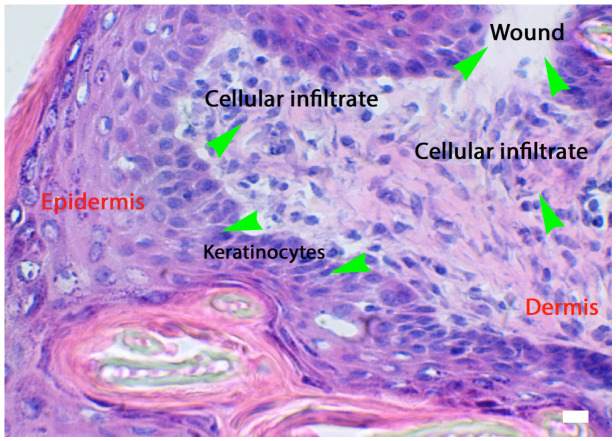
The activation of the skin microenvironment in conditions of injury such as a wound. The responses of keratinocytes and cellular infiltrate (see green arrows for respective locations). HE staining and light microscopy; 2D image, scale bar = 10 microns, [1].

Keratinocytes (KCs) play a crucial role in the wound-healing process, as seen in Figure 2 particularly during the inflammatory phase. However, a role during the hemostasis is postulated. These cells become activated, secreting a variety of cytokines and growth factors essential for healing. Approximately 24 h after injury, KCs begin to migrate toward the wound site. This migration involves detachment from the underlying basement membrane, a process primarily facilitated by MMP1, which is expressed abundantly at the edges of the wound. In contrast to resting basal cells, migrating basal keratinocytes upregulate the expression of CD44. The cellular response is further stimulated by IL8, which is highly expressed at the wound margin and promotes closure. During migration, keratinocytes express CXCR2, the receptor for IL8, and GROalpha [1,2,3]. The keratinocyte migration and viability increased after treatment with the growth factors IGF1 and keratinocyte growth factor (KGF) [24]. In addition, *FOSL1* can promote keratinocyte migration and proliferation via the IL17 signaling pathway, which is essential for epidermal wound healing. This mechanism is critical for successful epidermal restoration and plays a significant role in improving the wound healing process [25].

Their adhesion to a provisional matrix consisting of fibronectin and cross-linked fibrin is crucial for sustaining migration and healing.

**Figure 3:**
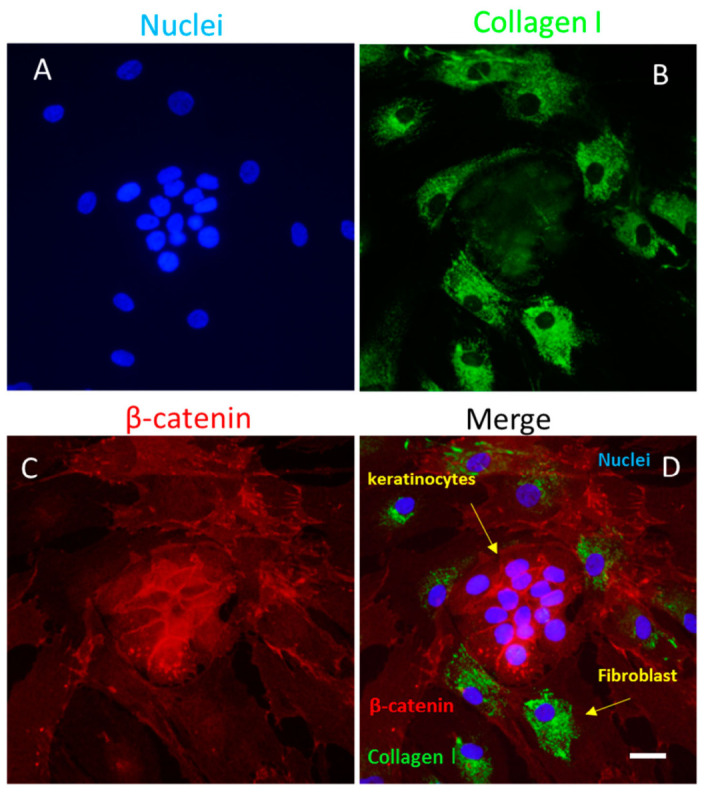
The co-culture of keratinocytes and fibroblasts stained for β-catenin (Alexa red) and collagen type I (Alexa green): (**A**) DAPI-stained nuclei; (**B**) fibroblasts labeled for collagen type I with Alexa green; (**C**) keratinocytes labeled for β-catenin with Alexa red; (**D**) merged image. 2D images (images are the results of the maximum projections of all zetastack), confocal microscopy, scale bar = 100 μm, [1].

While the mechanisms driving re-epithelialization remain partially elucidated, keratinocytes are known to modulate fibroblast activity by enhancing their proliferation (see Figure 3) and the secretion of growth factors that also encourage the proliferation of keratinocytes [1,2,3]. Additionally, keratinocytes affect the inflammatory environment through the recognition of Pathogen-Associated Molecular Patterns (PAMPs) and DAMPs, which activates pro-inflammatory signaling pathways. This process involves the production of various interleukins, including IL1, IL6, IL8, IL10, IL18, and IL20, along with TNFalpha and several chemokines such as RANTES, MCP1, and Macrophage Inflammatory Protein (MIP1). Lastly, keratinocytes influence the differentiation of fibroblasts into myofibroblasts, balancing the pro-inflammatory conditions with TGF-beta signaling, contributing to the overall healing process [1,2,3].

#### 3.3.2. Endothelial Cells

In the context of wound healing, endothelial cells play a crucial role during the proliferative phase by facilitating the recruitment of leukocytes from the bloodstream to injured tissues. This process is significantly influenced by various chemokines, such as MCP1, Regulated Upon Activation Normal T-Cell Expressed and Secreted (RANTES), IL8, GROalpha, IP10, and MIG, which contribute to angiogenesis—the formation of new blood vessels. The presence of the Glu-Leu-Arg (ELR) motif in these chemokines enhances their angiogenic potential, particularly noted in chemokines like GROalpha, GRObeta (CXCL2), and GROgamma (CXCL3), along with others including CTAPIII and beta-thromboglobulin. Conversely, CXC chemokines that lack the ELR motif, such as IP10 and MIG, are known to inhibit angiogenesis. MCP1 is especially important as its expression establishes a chemokine gradient that directs specific leukocytes to inflamed regions [1,2,3].

The process through which leukocytes infiltrate damaged areas is intricately linked to changes in blood vessel permeability, primarily regulated by VEGF. Other growth factors, such as FGF, PDGF, and Stromal-derived Growth Factor (SDF1), also influence endothelial cell proliferation and directional movement (chemotaxis). Notably, the activities of VEGF and FGF are modulated by proteoglycans, particularly the syndecans containing heparin sulfate [1,2,3].

Endothelial cell migration and proliferation involve complex reorganization of the cytoskeleton, alterations in adhesion molecules, and specific signal transduction pathways. The stabilization of angiogenesis ultimately requires the recruitment of pericytes, which support the structure of new blood vessels. Furthermore, the repair mechanisms of endothelial cells during angiogenesis are related to endothelial progenitor cells (EPCs). These progenitor cells possess markers characteristic of both endothelial cells and hematopoietic stem cells, such as CD146, VWF, and VEGFR2, serving as a source of pro-angiogenic cytokines that promote healing and tissue regeneration [1,2,3].

Lastly, fibroblast-derived extracellular vesicles seem improve angiogenesis and endothelial cell (EC) activity, suggesting that fibroblasts and ECs communicate positively for healing. Administration of extracellular vesicles (ECEVs) to healing mouse wounds resulted in a significant enhancement of collagen density and an increase in the quantity of fibroblasts present in the wound bed of scar tissue. This study reveals a previously unrecognized role of ECEVs in modulating fibroblast function, thereby contributing to the wound repair process [26].

#### 3.3.3. Pericytes

Pericytes represent a novel cell type essential to wound healing processes. These cells collaborate with endothelial cells during neo-angiogenesis and play a critical role in regulating the diameter of newly formed blood vessels. Research indicates that pericytes function synergistically with macrophages, especially during the inflammatory phase of wound healing. Furthermore, these cells appear to activate an immune response by stimulating T lymphocytes, facilitated by their secretion of various cytokines. Recent investigations have also revealed that pericytes possess the ability to differentiate into myofibroblasts through a mechanism mediated by Platelet-Derived Growth Factor (PDGF) [1,2,3].

#### 3.3.4. Fibroblasts

Fibroblasts are essential participants in the wound healing process, fulfilling various roles across different stages of healing. They secrete extracellular matrix components during the proliferative stage and contribute to remodeling efforts later on. In the early phases of wound repair, fibroblasts found in the reticular dermis are primarily active, while those in the papillary dermis are recruited during re-epithelialization. Importantly, fibroblasts are not a homogeneous cell type, as seen in Figure 2 which shows these cells in an in vitro model; rather, they comprise diverse subtypes that originate from different embryonic sources and are spatially distributed across distinct anatomical regions, including various layers of skin [27,28].

During the inflammatory phase of wound healing, fibroblasts play a pivotal role in orchestrating the local immune response through multiple pathways. They secrete pro-inflammatory cytokines and growth factors such as TNFalpha, IFNgamma, IL6, and IL12, in addition to releasing a variety of chemokines like CXCL1, CX3CL1, and CCL2. Furthermore, fibroblasts enhance cellular interactions by expressing adhesion molecules, including ICAM1 (CD54) and CD40, which stimulate dendritic cell activity and promote an effective immune response.

They also secrete MMPs, which are crucial for the stromal remodeling necessary for wound healing, as they break down extracellular matrix components to facilitate the migration and proliferation of fibroblasts during the healing process. The interplay between fibroblasts and macrophages is critical in transitioning from the inflammatory phase to the proliferative phase, thereby expediting the overall healing process [1,2,3].

As fibroblasts migrate and proliferate, they are driven by the secretion of growth factors such as PDGF, TGF-beta, FGF, and VEGF. In the proliferative phase, they are particularly active in promoting vascularization through the release of agents like VEGF, FGF, Angiopoietin-1 (ANG1), and Thrombospondin (TSP).

Recent research has highlighted that fibroblasts are targets for chemokines, including MCP1, which upregulate the expression of MMP1 and TIMP-1 in these cells. The synthesis of extracellular matrix by fibroblasts not only aids in re-epithelialization but also provides essential support for the functioning of myofibroblasts during the healing process [1,2,3].

#### 3.3.5. Dermal Sheath Cells

Dermal sheath cells (DSCs) are specialized mesenchyme-derived cells that establish a connective tissue layer surrounding hair follicles, which is essential for the processes of hair follicle regeneration and cycling. These cells are significant because they house progenitor cells capable of differentiating into various other cell types, including those of the dermal papilla. DSCs are important for both hair follicle function and wound healing because they act like fibroblasts and help repair tissue.

Furthermore, these cells possess contractile properties that facilitate the movement of hair follicles throughout the hair growth cycle, highlighting their multifaceted contributions to both hair biology and healing processes [29].

### 3.4. Phases: Maturation and Remodeling

#### Myofibroblasts

Granulation tissue that develops post-injury experiences contraction mostly driven by myofibroblasts, which originate from fibroblasts at the damage site. Myofibroblasts are essential in wound contraction and tissue remodeling by assuming a contractile phenotype, principally influenced by actin, and by secreting extracellular matrix components. The differentiation of fibroblasts into myofibroblasts is modulated by TGFbeta1 inside the skin’s milieu, as well as by the mechanical characteristics of the extracellular matrix (ECM). The transformation of collagen type III to collagen type I influences the tensile strength of the extracellular matrix, thus affecting fibroblast activity. Upon restoration of tissue integrity, the activity of myofibroblasts declines, resulting in a decrease in their number via apoptotic mechanisms. Nonetheless, the regulatory mechanisms, including the signals and timing related to these occurrences, are not fully understood [27,28].

## 4. Inconsistencies in Understanding Some Behavior of the Key Cells Involved in Wound Healing

Current understanding of wound healing, while comprehensive regarding basic phases (hemostasis, inflammation, proliferation, and remodeling), contains significant inconsistencies and knowledge gaps that prevent effective treatment of chronic wounds and pathological scarring.

Macrophage polarization, switching from pro-inflammatory (M1) to anti-inflammatory (M2), is a critical but inconsistent process in wound healing; excessive M2 persistence can hinder the repair of diabetic wounds. The role of fibroblast heterogeneity is unclear, particularly why some fibroblasts become hyperactive and lead to excessive fibrosis, while others do not adequately deposit collagen. Angiogenesis regulation is inconsistent; diabetic wounds often lack sufficient VEGF for angiogenesis, while chronic venous stasis ulcers have high VEGF but still do not heal effectively. Additional studies are necessary to uncover the signals that drive M1-to-M2 transitions, clarify the roles of different fibroblast sub-populations in scarring versus regeneration, and understand the resistance of wound environments to angiogenic stimuli [30].

## 5. Neuroimmunomodulation During Wound Healing

It is known that the interaction between the neurological system and the immune system emphasizes its crucial function in several physiological processes, especially in wound healing. Particular trials demonstrate the impact of neurogenic stimuli on wound healing after damage. The surgical excision of cutaneous nerves in animal models results in delayed wound healing, highlighting the vital connection between the neurological and immune systems in tissue repair and recovery [3,31,32,33,34,35,36].

### 5.1. Inflammatory Phase

**Figure 4:**
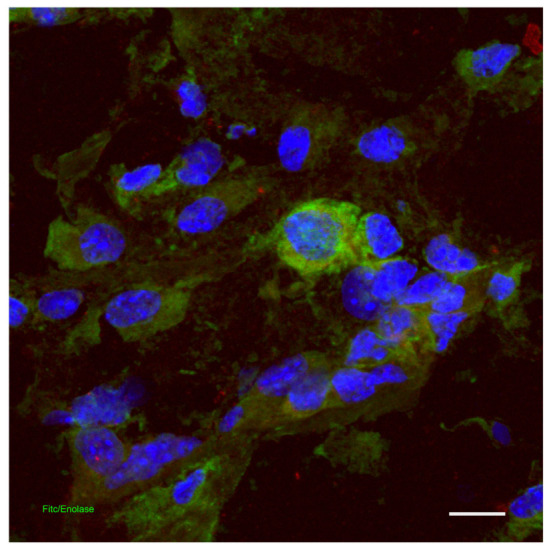
Scattered distribution of neuronal cells in the dermis of a wound. Double colocalization of Enolase (green) and DAPI (blue). 2D images (images are the results of the maximum projections of all zetastack), confocal microscopy scale bar = 100 microns [37].

In the inflammatory phase of wound healing, neuropeptides are essential in regulating the immune response and facilitating tissue restoration. Substance P (SP), a crucial neuropeptide secreted by cutaneous neurons (Figure 4), increases microvascular permeability and vasodilation by facilitating nitric oxide release and modulating endothelial cell activity. Furthermore, SP promotes the activation of adhesion molecules and monocyte chemotaxis while enhancing the activity of inflammatory cells. It is essential in modulating the production and secretion of pro-inflammatory cytokines, including interleukins, TGFalpha, and TNFalpha [3,31,32,33,34,35,36].

Neutral endopeptidase (NEP) modulates the activity of SP by inhibiting it and competing with the neurokinin-1 receptor (NK-1R), which alters inflammatory signaling mechanisms during WH. Neurokinin A (NKA), another important neuropeptide, is produced upon damage and activates cutaneous target cells, such as keratinocytes and dermal endothelial cells, predominantly via the neurokinin-2 receptor (NK-2R), thus further influencing the control of skin inflammation [3,31,32,33,34,35,36].

Corticotropin-releasing hormone (CRH), detected in cutaneous sensory neurons using immunohistochemical analysis, triggers mast cell degranulation and functions as a pro-inflammatory mediator, enhancing vascular permeability and promoting the production of pro-inflammatory cytokines. CRH is also involved in facilitating angiogenesis in the epidermis [3,31,32,33,34,35,36].

Calcitonin gene-related peptide (CGRP) functions as a vasodilator that enhances angiogenesis and promotes plasma extravasation, intensifying the inflammatory reactions initiated by other mediators such as SP. Activin, a member of the TGFβ superfamily, enhances CGRP production in sensory neurons, especially post-injury, underscoring its significance in wound healing [3,31,32,33,34,35,36].

Nerve growth factor (NGF) is essential during the inflammatory phase since it promotes the release of CGRP from peripheral nerve terminals into adjacent tissues. Neuropeptide Y (NPY) and CGRP have both pro-inflammatory and anti-inflammatory properties in murine model investigations, highlighting the complexity of neuropeptides in inflammatory reactions [3,31,32,33,34,35,36].

The significance of nitric oxide (NO) as an extracellular chemical messenger in wound healing has been acknowledged. Nitric oxide synthase (NOS) catalyzes the formation of NO, which is activated by diverse inflammatory mediators, apoptotic debris, or bacterial constituents. Inducible nitric oxide synthase (iNOS) plays a role in the inflammatory phase of wound healing by facilitating vasodilation and exhibiting antibacterial properties. The complex interaction between these neuropeptides and inflammatory mediators highlights their significance in wound healing [3,31,32,33,34,35,36].

### 5.2. Proliferative Phase

SP induces DNA synthesis, resulting in notable proliferation in fibroblasts, keratinocytes, and endothelial cells, while also facilitating angiogenesis, perhaps via NO. It is essential for the remodeling of granulation tissue, promoting the proliferation and migration of dermal fibroblasts, and augmenting the production of epidermal growth factor and its receptor [3,31,32,33,34,35,36].

Neurotrophins, such as nerve growth factor NGF, are present in central and peripheral neurons, along with diverse cell types including fibroblasts and keratinocytes. They are crucial for the survival and functionality of sensory and autonomic neurons and have anti-inflammatory effects. NGF has shown the ability to promote the proliferation of immature local cells in lesions, boost angiogenesis, and stimulate neurite outgrowth, supported by data from both animal research and human instances that indicate its role in epithelial healing. Moreover, neurokinin A induces the release of NGF in the epidermis [3,31,32,33,34,35,36].

Additional neuropeptides implicated in the proliferative phase include GRP, CGRP, galanin, vasoactive intestinal peptide (VIP), and pituitary adenylate cyclase-activating peptide (PACAP). CGRP, present in the central and peripheral nerve systems, may facilitate angiogenesis and keratinocyte functions; however, its overall efficiency in wound healing remains unknown [3,31,32,33,34,35,36].

Galanin, secreted by afferent neurons, has been shown to transmit signals via G-protein coupled receptors and has anti-proliferative properties; however, it may also enhance NGF upregulation in in vitro environments. VIP acts as a growth factor that affects keratinocyte behavior, promotes histamine production to trigger vasodilation, and improves reinnervation after nerve injury, as shown in rat studies [3,31,32,33,34,35,36].

Furthermore, SP, CGRP, and VIP are recognized for their modulation of matrix metalloproteinase activities, which influence collagen synthesis in the process of cutaneous wound healing. PACAP acts as a vasodilator in sensory cutaneous nerves, produced after neural activation. This leads to vasodilation and skin inflammation, which in turn promotes keratinocyte growth and triggers histamine release from mast cells [3,31,32,33,34,35,36].

### 5.3. Remodeling Phase

The regeneration of sensory nerve fibers in the healed dermis and epidermis is related to the remodeling stage of cutaneous wound healing.

SP is essential in this process since it stimulates the synthesis of NGF from human dermal microvascular EC, which is vital for nerve fiber regeneration after cutaneous damage.

NGF is thought to promote tissue remodeling, underscoring its significance in the healing process. Furthermore, sensory and sympathetic nerves produce NT3, an essential neurotrophic growth factor required for nerve development and maintenance.

The brain-derived neurotrophic factor (BDNF) is crucial for the survival and functional maturation of sensory neurons, with keratinocytes, fibroblasts, and myofibroblasts producing both BDNF and its receptors, so facilitating their proliferation and differentiation.

The interplay of neuropeptides such as SP, CGRP, and VIP seems to affect MMP activity and the production of collagen types I and III during cutaneous wound healing.

Notwithstanding these insights, the precise functions of neuropeptides and cutaneous innervation throughout the remodeling phase are still inadequately comprehended. Moreover, Protein Gene Product 9.5 (PGP 9.5) is produced by fibroblasts throughout the granulation tissue and remodeling stages, highlighting the complex cellular interactions in wound healing [3,31,32,33,34,35,36].

## 6. Molecular Events in Wound Healing

### 6.1. Growth Factors, Cytokines, and Other Substances

The process of wound healing is complex and mostly governed by many molecules, such as cytokines and growth factors, which are produced during distinct phases of healing. The regulation of these processes is essential, since any changes might impede normal healing and possibly result in chronic wounds. Key pro-inflammatory cytokines such as IL1beta, TNFalpha, and IL6 recruit inflammatory cells to the wound location. The inflammatory cells then release growth factors, including PDGF and TGFbeta, which attract proliferating fibroblasts to the lesion [32,38,39,40].

Additionally, macrophages and activated fibroblasts synthesize various growth factors, such as FGF2, KGF, FGF7, EGF, Hepatocyte Growth Factor (HGF), TGFalpha, and IGF1, to facilitate epithelialization. VEGF and PDGF, secreted by fibroblasts, keratinocytes, and macrophages, activate endothelial cells, therefore commencing angiogenesis [32,38,39,40].

Factors influencing wound healing encompass transcription factors (notably the E2F family), diverse signaling pathways (including the Wnt/beta-catenin pathway), and molecules such as Signal Transducer and Activator of Transcription 3 (STAT3), homeobox genes, hormone receptors (for androgens, estrogens, and glucocorticoids), Peroxisome Proliferator-Activated Receptors (PPARs), Activator Protein 1 (AP1), c-Myc, and ETS-related Gene 1 (Erg1), in addition to proteases (specifically MMPs), cytoskeletal proteins, and enzymes that modulate cellular redox balance. These constituents are interconnected, collectively contributing to the release of bioactive factors vital for wound healing [32,38,39,40].

Invertebrates and vertebrates use unique transcription-independent diffusible signals during wound healing; notably, hydrogen peroxide and adenosine, which facilitate further autocrine ATP release, are essential components. Protein Kinase C (PKC), Ca^2+^/Calmodulin-dependent Protein Kinase 4 (CaMK4), and reactive oxygen species (ROS) modulate genetic transcription subsequent to the rapid elevation of intracellular calcium concentration, which is crucial for cellular communication, migration, adhesion, inflammatory responses, angiogenesis, and re-epithelialization [32,38,39,40].

Tissue injury triggers Ca^2+^ waves that activate RHO family GTPases, promoting actin polymerization and actomyosin contractility, therefore preserving stromal integrity. Increased intracellular calcium concentrations subsequently activate many signaling pathways, including c-Jun N-terminal kinase (JNK) and Mitogen-Activated Protein Kinase (MAPK), resulting in the activation of transcription factors and the overexpression of cytoskeleton-related insult-response genes. The production of ATP and its subsequent activation via purinergic receptors are crucial in the healing process, since they elicit responses in nearby healthy epithelial cells that detect DNA damage. This initiates the synthesis of certain growth factors such as EGF, which activate many pathways essential for effective wound healing [32,38,39,40].

### 6.2. Genetic Activation in Wound Healing

Wound repair is regulated by a complex genetic network integrating growth factors, cytokines, ECM, and cellular signaling pathways. Canonical regulators, such as TGFbeta, VEGF, FGF, and MMP, coordinate the interactions among keratinocytes, fibroblasts, endothelial cells, and immune populations. However, these pathways are modulated by underlying genetic variation—both rare, high-penetrance mutations and common polymorphisms—that influence repair outcomes [41].

The phases of wound healing are affected by genes that encode cytokines, chemokines, and growth factors, exhibiting considerable functional overlap. During the earliest healing stages, a significant gene expression pattern is seen, highlighting several highly differentially expressed genes (DEGs). Principal hub genes include Tyrosinase (TYR), Tyrosinase-Related Protein 1 (TYRP1), and Dopachrome Tautomerase (DCT), which are vital for melanin synthesis. In the inflammatory and proliferative stages, 85 differentially expressed genes (DEGs) and 164 downregulated proteins have been discovered, including checkpoint genes such as Checkpoint Kinase 1 (CHEK1), Cyclin B1 (CCNB1), and Cyclin-dependent Kinase 1 (CDK1), which are essential to the cell cycle and the P53 signaling pathway. The remodeling phase reveals 121 differentially expressed genes (DEGs) and 49 weakly expressed genes, with hub genes including Collagen Type Alpha Chain 1 (COL4A1), Collagen Type 4 Alpha Chain 2 (COL4A2), and Collagen Type 6 Alpha Chain 1 (COL6A1) associated with protein digestion and ECM receptor interactions [39,40,41].

Research has identified significant cytokines (e.g., IL1beta, IL6, CCL4) and their function in wound healing, especially in modulating skin repair and keratinocyte motility. A deficit in certain chemokines, including CXCL1 and CXCL5, has been associated with compromised healing in experimental animals. Pro-inflammatory genes (TNFalpha, IFNgamma, TGFbeta) are promptly activated post-injury, subsequently including genes (VEGF, PDGF, FGF2, MMP) that promote fibroblast and keratinocyte activity, therefore facilitating epithelialization and angiogenesis throughout the healing process. The remodeling phase involves the stimulation of TGFbeta1 and MMP to promote collagen production and extracellular matrix resorption [39,40,41].

Epigenetic mechanisms play a pivotal role in wound repair by influencing gene expression and cellular functions through various mechanisms, including DNA methylation, histone modifications, non-coding RNA (ncRNA) regulation, and RNA methylation. In the hemostasis phase of wound healing, DNA methylation affects genes like platelet endothelial aggregation receptor 1, thereby influencing platelet function. Concurrently, histone modifications such as methylation and acetylation are crucial for modulating inflammatory responses and activating fibroblasts, which are essential for tissue repair. Non-coding RNAs, particularly microRNAs and long ncRNAs, further contribute to wound healing by managing processes related to cell proliferation, collagen deposition, and scar formation. RNA methylation, specifically N6-methyladenosine modifications, plays a significant role in regulating autophagy and fibrosis through interactions with YTH domain family proteins, which suggests a complex interplay among different epigenetic factors during the wound healing process. The understanding of these key epigenetic regulators offers insight into potential therapeutic strategies for improving wound healing outcomes. Despite this promise, translating epigenetic research into clinical applications results in major complications as a result of the intricacies of epigenetic networks and the requirement for precise regulatory tools. Future research should concentrate on delineating cell-specific and spatiotemporal regulatory mechanisms of epigenetic modifications in wound healing, with the aim of identifying therapeutic targets to minimize scar formation and avert chronic wounds [39,40,41,42].

Research demonstrates heterogeneity in wound healing across people, as seen by mice models that have differing healing capacities across several strains. The MRL/MpJ-Faslpr (MRLF) mouse lineage exhibits accelerated healing compared to the delayed healing seen in the C57BL/6 and SJLJ strains. The genetic effect designates MRLF mice as an important model for investigating mammalian wound healing [41].

## 7. Influence of the Microbiota on Wound Healing

It is becoming more well recognized that wound healing involves a host-microbe interaction that influences angiogenesis, tissue regeneration, and inflammation.

Platelets and keratinocytes function as innate immune sensors during the hemostasis phase. When triggered by *Staphylococcus epidermidis*, keratinocytes produce pattern recognition receptors (PRRs) such as TLR2, which react to staphylococcal lipoteichoic acid (LTA) and promote interleukin and defensin responses. Although more study is needed to understand the direct signaling pathways involving platelets and keratinocytes, platelets also react to Gram-positive ligands. On the other hand, lipopolysaccharide (LPS) from *Pseudomonas aeruginosa* may activate TLR4, increasing inflammation and compromising clot integrity and healing [43].

While pathogenic strains maintain chronic inflammation via quorum-sensing mechanisms that promote biofilm formation, commensal bacteria like *S. epidermidis* facilitate the resolution of inflammation by producing IL-10 and TGF-β. Biofilms provide hypoxic environments that prolong inflammation in chronic wounds by impeding appropriate immune responses while sustaining VEGF synthesis [43].

Through GPR41/43 engagement, microbial metabolites (short-chain fatty acids) improve fibroblast activities and extracellular matrix (ECM) rebuilding during the proliferation phase. This reciprocal interaction between the host and microbial populations may significantly influence the mechanics of wound healing [43].

The microbiota indirectly influences healing during the remodeling stage. While other microbial taxa modify lipid levels that impact the skin barrier and ECM organization, *S. epidermidis* may strengthen antimicrobial defenses. In the meantime, *P. aeruginosa* generates proteases that harm collagen and hinder the healing process. Therefore, commensal microorganisms work together to improve defensive systems and the matrix against the disruptive effects of infections [43].

According to recent research, good wound healing is associated with a return to commensal microbial dominance, underscoring the need for microbiome re-equilibration for a full recovery.

### Microbiota and Chronic Wounds

Next-generation sequencing techniques, especially 16S ribosomal RNA (rRNA) gene and metagenomic sequencing, have become crucial in personalizing wound care, overcoming the limitations of traditional culture methods that frequently underestimate bacterial load. A thorough investigation of 2963 chronic wound samples demonstrated a prevalence of Staphylococcus and Pseudomonas species, along with pathogenic anaerobes, regardless of the lesion’s etiology. A negative connection was observed between the prevalence of Corynebacterium and Streptococcus in chronic wounds of individuals suffering from severe pain. A significant prevalence of Streptococcus epidermidis was observed; this skin commensal, contingent upon the strain, had the capability to build biofilms and impede wound healing. Conversely, Alcaligenes faecalis was associated with decreased wound severity and improved healing, questioning the traditional dependence on empirical antibiotics [43,44,45,46].

Research highlighting the complex structure of chronic wound microbiota indicates that comprehending microbial makeup might guide tailored treatment approaches, moving away from generalized antibiotic use. Kits based on polymerase chain reaction have been created for bacterial identification and the detection of antibiotic-resistant indicators; nevertheless, their efficacy in clinical environments requires confirmation via randomized controlled trials [43,44,45,46].

Furthermore, investigations into the skin microbiome of individuals with and without *hidradenitis suppurativa* (HS) have revealed that HS lesions are primarily inhabited by *Corynebacterium* and *Porphyromonas*/*Peptoniphilus* species, which are absent in healthy controls, suggesting their possible involvement in HS pathogenesis. The concentration of anaerobic bacteria in HS may clarify the efficacy of ertapenem therapy in severe instances [43,44,45,46].

Moreover, research has shown that commensal microorganisms might enhance healing by augmenting the synthesis of antimicrobial peptides (AMPs). *S. epidermidis* promotes the production of antimicrobial peptides to combat infections induced by Streptococcus and *S. aureus*. This highlights the therapeutic potential of the skin microbiome in both infection prevention and tissue regeneration, underscoring its significance in wound care and healing processes [43,44,45,46].

## 8. Alterations in the Normal Wound Healing Process

Chronic skin lesions are characterized as wounds that fail to heal within six to eight weeks, resulting from ongoing inflammatory responses that interfere with the standard healing process. These lesions can arise from numerous underlying diseases, with estimates suggesting 1406 different clinical presentations influenced by high comorbidity rates, especially in the elderly. Key contributors to chronic ulcer development include elevated protease activity, which affects growth factor function and tissue regeneration, alongside increased levels of MMPs and reduced TIMPs. This imbalance fosters excessive tissue degradation rather than repair. Cathepsin D, a protease important in wound healing, can impair recovery if improperly regulated [47]. Effective healing relies on the delicate balance of new tissue production and the breakdown of the EC, yet this equilibrium is often compromised in chronic wounds due to factors such as malnutrition, diabetes, and corticosteroid usage, leading to lowered nitric oxide levels and fibrin sheath formation. Chronic wounds share mechanisms with acute wounds but exhibit dysregulation characterized by excessive neutrophil presence, resulting in chronic inflammation and increased MMP release. These neutrophils exacerbate tissue injury and reduce key repair proteins like PDGF and TGF-beta, with keratinocytes showing incomplete activations of proliferative genes and a poor response to migratory signals. Infections further exacerbate chronic wounds by escalating bacterial loads, prolonging inflammation, and enhancing MMP synthesis, while biofilms obstruct effective treatment and promote chronic ulceration through continuous bacterial colonization and inflammatory mediator release [32,48].

**Keloids**, another type of chronic skin lesion, represent excessive fibrous tissue growth beyond the original wound site, leading to various complications, including pain, itching, and psychosocial issues. The formation of keloids involves an overproduction of ECM components by fibroblasts and is influenced by the immune microenvironment, marked by increased immune cell infiltration compared to normal skin.

Macrophages, particularly the M2 subtype, play a significant role by modulating the healing environment and contributing to fibrosis, which can exacerbate the formation of keloids and lead to complications such as pain and psychosocial issues.

The microbial profile of keloids varies, with active keloids displaying higher levels of *Acinetobacter* and *Pseudomonas* compared to normal skin, while inactive keloids resemble normal microbial profiles [48,49].

**Hypertrophic scars**, commonly seen in burn survivors, are characterized by raised and inflexible tissue that can lead to functional limitations and aesthetic concerns. Their development involves pathological ECM production driven by fibroblasts, prolonged inflammation, and abnormal neovascularization. Multiple cell types, including macrophages, keratinocytes, and MC, significantly influence fibroblast activities, leading to excessive matrix synthesis [48].

Fifteen percent of people with Type 2 Diabetes Mellitus (T2DM) have chronic diabetic wounds, especially diabetic foot ulcers (DFUs). These wounds can cause serious problems, such as amputations.

In diabetic patients, healing is impeded by prolonged inflammation and impaired epithelialization, as evidenced by a heightened neutrophil response and a disrupted balance of regulatory immune responses. Treg lymphocyte migration is often inhibited while inflammatory Th17 cells dominate, exacerbating neutrophilic inflammation [50]. During the proliferative phase, skin cell proliferation decreases, and fibroblasts exhibit inactivation, resulting in reduced matrix deposition [51,52]. This is further complicated by increased oxidative stress, angiogenic deficiencies, and a shift from M1 to pro-inflammatory M2 macrophage polarization, mediated by exosomes, ultimately obstructing the healing process in diabetic wounds [52].

## 9. Advanced Therapies in Wound Healing

Wound care is a significant issue in medicine, and innovative therapies have been proposed to address this problem.

Stem cell therapy leverages the regenerative capabilities of stem cells to address tissue damage through repair and replacement. These stem cells, which can be sourced from adipose tissue or bone marrow, possess the ability to differentiate into various cell types and release growth factors that facilitate angiogenesis and tissue regeneration. Recent research highlights the effectiveness of adipose-derived mesenchymal stem cells (AD-MSCs) specifically in diabetic wound models, demonstrating improvements in cell proliferation, angiogenesis, collagen deposition, and a decrease in inflammation.

However, stem cell therapy is not without its challenges; issues related to standardization and scalability must be resolved to enable its wider clinical adoption [40], particularly in ensuring consistent quality and efficacy across different sources of stem cells.

Drugs on wound healing include anticoagulants, antimicrobials, angiogenesis agents, antineoplastic drugs, anti-rheumatoid drugs, nicotine, steroids, vasoconstrictors, and non-steroidal anti-inflammatory drugs (NSAIDs) [53]. Glucagon-Like Peptide-1 (GLP1) is an intestinal insulinotropic peptide that accounts for about 60% of postprandial insulin production; Exendin 4 (Exe4) has 53% similarity with GLP1, making it a promising option. There are some indications from experiments that Exe4 may have a function in facilitating tissue regeneration [51].

Phototherapy is becoming increasingly important in wound healing, with lasers being crucial in assisted healing.

Photodynamic therapy (PDT) uses harmless light in the protoporphyrin absorption spectrum with non-toxic photosensitizing dyes [54].

PDT has been explored for its ability to reduce all types of microorganisms by inducing ROS without inducing resistance to conventional antibiotics. However, owing to the lack of published research and the necessity for several repeated sessions with the lights and photosensitizers that are now available, the use of PDT for aided wound healing is not yet common in clinical practice [55].

Low-level laser light therapy (LLLT) induces cellular modification, leading to beneficial clinical results. Light-emitting diodes (LEDs) have been promoted to simplify the use of the technique. LEDs provide targeted biofilm control and tend to reduce the inflammatory state of the progressing lesion. Among the more encouraging adjuvant treatments, electrical stimulation stands out, with some clinical trials demonstrating the utility of electric field stimulation in wound healing. Ultrasound has also been used as a promising treatment [56].

Recent advancements in wound healing encompass several innovative approaches, including nano-fibrous scaffolds with anti-inflammatory properties, metal–organic frameworks (MOFs), isoxazole derivatives, and LED-activated graphene oxide compounds. Exosome-based therapies signify progress in regenerative treatment, while new culture systems mimicking tissue mechanical tension offer better monitoring of the healing process. A significant breakthrough in regenerative medicine is bioprinting, utilizing 3D printers with bio-inks from living cells to create personalized skin grafts and tissue scaffolds, enhancing acceptance and reducing healing times. These bioprinted grafts are under investigation for treating severe burns and chronic wounds, capable of replicating the natural skin’s multi-layered structure for both functional and aesthetic restoration. Research institutions and biotech companies are piloting this technology in wound care centers, showing promising early results. Additionally, biophysical methods like shock wave therapy, ultrasound, and electrical stimulation enhance cellular activity and tissue repair. AI-driven digital imaging tools are improving bacterial detection and monitoring healing progress, leading to more targeted treatments and less dependence on antibiotics. Advanced wound dressings, including smart dressings and nanotechnology-based therapeutics, enable controlled delivery of therapeutic agents, significantly improving healing outcomes [40,57].

## 10. Current Limitations in the Study of Wound Healing

The process of wound healing, while recognized in medical practice for years, remains partially understood. Current wound healing research often underestimates the critical role of the skin microenvironment and extracellular matrix in wound healing. The dynamic reciprocity of the wound microenvironment, characterized by continuous bidirectional interactions between cells and their surrounding environment, is crucial. Cell-extracellular matrix interactions not only direct and regulate cell morphology but also influence cell differentiation, migration, proliferation, and survival during tissue development, such as embryogenesis and angiogenesis, as well as during pathological processes including cancer, diabetes, hypertension, and chronic wound healing [5,14]. It follows that the influence of the skin microenvironment is crucial for wound healing, as it includes the control of inflammation at the transition from inflammation to repair; the utilization of the extracellular matrix scaffold, which provides structure and regulatory signals for cellular activity; the activation of stem cells, driven by signals from the injured area for re-epithelialization; the interaction with the microbiome, where beneficial bacteria strengthen immunity while pathogenic ones can hinder healing; and physical factors, where a moist and slightly acidic environment promotes faster healing than dry conditions. A balanced microenvironment is essential for effective wound healing [58].

In addition, research on wound healing indicates that conventional descriptive models of the four stages (hemostasis, inflammation, proliferation, and remodeling) often do not adequately represent the intricacies of non-linear and chronic healing processes. Contemporary research challenges the inflexible, linear phase model, highlighting that these processes are ongoing and significantly interrelated.

The disparity in biomarker translation is a pressing issue, since there is an absence of economical, point-of-care diagnostic instruments for molecular biomarkers.

Conventional animal models face growing criticism for their inability to correctly replicate human skin architecture and immunological responses, resulting in a “translational gap” where laboratory results do not translate to human treatment trials [32,59].

## 11. Actual Perspectives in the Study of Wound Healing

Recent research has primarily focused on the cellular constituents of healing, highlighting novel cell types like MCs, T Regs, MC, and DSCs. In addition to cellular studies, molecular aspects, particularly genetics, continue to be explored. There is growing interest in neuro-immunomodulation, leading to the discovery of various neural mediators crucial for healing. The activation of microbiota during wound healing is also essential for a deeper understanding of the process. In particular, exploring future possibilities such as predictive microbiome signatures or targeted microbiome modulation might be helpful in clinical practice.

Thus, it is crucial for future research to integrate fields of study such as microbiome interactions, neuro-immune signaling, and genetics to create a cohesive framework.

Innovative imaging technologies, such as confocal and multiphoton microscopy, coupled with machine learning, have advanced this field, facilitating detailed cellular process analyses and creating predictive models that clarify the intricacies of wound repair while minimizing animal usage.

Anticipated developments should include the incorporation of artificial intelligence (AI) for the analysis of wound imagery and biomarker data, intelligent and bioactive therapies, customized wound care, and comprehensive integration.

These developments seek to overcome the constraints of conventional descriptive models and enhance comprehension of wound healing mechanisms [2,3,32,59].

## Data Availability

The original contributions presented in this study are included in the article. Further inquiries can be directed to the corresponding author.

## Abbreviations

Denomination	Acronym
Activator Protein 1	AP1
Adipose-Derived Mesenchymal Stem Cell	ADMSC
Amphiregulin	AREG
Angiopoietin 1	ANG1
Antimicrobial Peptide	AMP
Brain-Derived Neurotrophic Factor	BDNF
c-Jun N-Terminal Kinase	JNK
Ca^2+^/Calmodulin-Dependent Protein Kinase	CaMK
Calcitonin-Gene-Related Peptide	CGRP
Checkpoint Kinase 1	CHEK1
Chemokine (C-C motif) Ligand	CCL
Chemokine (C-C motif) Receptor	CXCR
Chemokine (C-X-C motif) Ligand	CXCL
Chronic Wound	CW
Collagen Type Alpha Chain	COL4A
Connective Tissue Chemokine-Activating Peptide	CTAPIII
Corticotropin-Releasing Hormone	CRH
Cyclin B1	CCNB1
Cyclin-Dependent Kinase 1	CDK1
Damage-Associated Molecular Patterns	DAMPs
Dendritic Cell	DC
Dermal Sheath Cells	DSCs
Differentially Expressed Genes	DEGs
Dopachrome Tautomerase	DCT
Endothelial Cells	EC
Endothelial Progenitor Cells	EPC
Epidermal Growth Factor	EGF
ETS-Related Gene 1	Erg1
Exendin 4	Exe4
Extracellular Matrix	ECM
Extracellular Vesicles	ECEVs
Fibroblast Growth Factor	FGF
Glucagon-Like Peptide-1	GLP1
Glycine-Related Peptide	GRP
Growth Arrest-Specific 5	GAS-5
Growth-Related Oncogene	GRO
Hepatocyte Growth Factor	HGF
Hidradenitis Suppurativa	HS
Human ASCs	hASCs
Interleukin	IL
Inducible Nitric Oxide Synthase	iNOS
Insulin-Like Growth Factor 1	IGF-1
Interferon	IFN
Interferon-g-inducible Protein 10	IP10
Keratinocyte Growth Factor	KGF
Keratinocyte	KC
Light-Emitting Diode	LED
Low-Level Laser Light Therapy	LLLT
Macrophage Inflammatory Protein	MIP
Macrophage-Derived Chemokine	MDC
Mast Cell	MC
Matrix Metalloproteinase	MMP
Mesenchymal Stem Cells	MSCs
Monocyte Chemoattractant Protein	MCP
Metal–Organic Frameworks	MOFs
Metastasis-Associated Lung Adenocarcinoma Transcript 1	MALAT-1
Mitogen-Activated Protein Kinase	MAPK
Monokine Induced by Gamma Interferon	MIG
Nerve Growth Factor	NGF
Neurokinin A	NKA
Neurokinin-1 Receptor	NK-1R
Neurokinin-2 Receptor	NK2R
Neuropeptide Y	NPY
Neurotrophin-3	NT3
Neutral Endopeptidase	NEP
Neutrophil Extracellular Traps	NET
Neutrophil-Activating Peptide 2	NAP2
Nitric Oxide	NO
Nitric Oxide Synthase	NOS
Non-Steroidal Anti-Inflammatory Drugs	NSAIDS
Pathogen-Associated Molecular Patterns	PAMPs
Pattern Recognition Receptors	PRR
Peroxisome Proliferator-Activated Receptors	PPARs
Photodynamic Therapy	PDT
Pituitary Adenylate Cyclase-Activating Peptide	PACAP
Plasmacytoid Dendritic Cells	PDCs
Platelet-Derived Growth Factor	PDGF
Prostaglandin E2	PGE2
Protein Gene Product 9.5	PGP 9.5
Protein Kinase C	PKC
Reactive Oxygen Species	ROS
Regulated Upon Activation Normal T-Cell Expressed and Secreted	RANTES
Regulatory T cells	T reg
Ribosomal RNA	RNAr
Signal Transducer and Activator of Transcription 3	STAT 3
Stromal-Derived Growth Factor	SDF
Substance P	SP
Thrombospondin	TSP
Tissue Inhibitor of Metalloproteinases	TIMPs
Toll-Like Receptor	TLR
Transforming Growth Factor	TGF
Tumor Necrosis Factor	TNF
Type 2 Diabetes Mellitus	T2DM
Tyrosinase	TYR
Tyrosinase-Related Protein 1	TYRP1
Vascular Endothelial Growth Factor	VEGF
Vasoactive Intestinal Peptide	VIP
Von Willebrand Factor	vWF
Wound Healing	WH
